# Effect of Allicin against Ischemia/Hypoxia-Induced H9c2 Myoblast Apoptosis via eNOS/NO Pathway-Mediated Antioxidant Activity

**DOI:** 10.1155/2018/3207973

**Published:** 2018-04-16

**Authors:** Lina Ma, Shangke Chen, Shaochun Li, Lijuan Deng, Yikui Li, Hao Li

**Affiliations:** ^1^Xiyuan Hospital, China Academy of Chinese Medical Sciences, Beijing, China; ^2^Xinjiang Ailexin Pharmaceutical Co., Ltd., Urumqi, Xinjiang, China; ^3^School of Basic Medical Sciences, Hebei University, Baoding, Hebei, China

## Abstract

Allicin (2-propene-1-sulfinothioic acid* S*-2-propenyl ester, diallyl thiosulfinate) is the main biologically active ingredient in garlic. The present study investigated the protective effect of allicin against cardiomyocyte apoptosis that was induced by ischemia* in vitro* and the potential molecular mechanisms that were involved in this antiapoptotic effect. The results indicated that allicin increased H9c2 cell activity and attenuated the rate of apoptosis that was induced by ischemia/hypoxia. Intracellular calcium concentrations significantly decreased in the allicin-treated groups. Bax expression significantly decreased, and Bcl-2 expression increased in allicin-treated rats. Nitric oxide blockade significantly inhibited these effects. Allicin also increased the activity of SOD and NO release and decreased MDA levels. Allicin significantly increased the expression of eNOS, Nrf2, and HO-1 proteins. Collectively, these findings demonstrate that allicin protects H9c2 cells against apoptosis, and this protective effect appears to occur via eNOS/NO pathway-mediated antioxidant activity.

## 1. Introduction

Ischemic heart disease (IHD) is one of the leading causes of global mortality, thus resulting in substantial social burdens worldwide [1–3]. Several studies have suggested that apoptosis occurs during the overall pathophysiological process of ischemia and plays an important role in the progression of cardiovascular diseases [4, 5]. Apoptosis is a pivotal form of cell death after acute myocardial infarction, which leads to abnormal loading conditions and, further, left ventricular dilatation and consequently symptomatic heart failure [6, 7]. Therefore, the inhibition of apoptosis may be an effective way to prevent heart failure and improve cardiac function. Numerous cardiovascular protective compounds have been discovered to limit the ischemic damage, but no satisfactory drug treatments have been developed for clinical practice.

Natural antioxidants from food sources have been used to prevent and treat IHD. Garlic has attracted particular attention because of its beneficial cardiovascular effects [8]. Allicin (2-propene-1-sulfinothioic acid* S*-2-propenyl ester, diallyl thiosulfinate) is the main biologically active ingredient in garlic. Allicin has been shown to exert a wide range of biological effects, including antioxidant activity, the inhibition of platelet activation, antitumor activity, and antimicrobial activity [9–12]. Our previous studies in rats found that allicin mitigates ischemic injury via inhibiting apoptosis [13]. It was also shown to prevent against myocardial apoptosis and fibrosis in a streptozotocin-induced diabetic models in rats [14]. Although abundant evidence supports a link between allicin and cardioprotection, the precise mechanisms by which allicin prevents IHD remain largely unknown. Therefore, the present study evaluated the protective effects of allicin against cardiomyocyte apoptosis that was induced by ischemia* in vitro* and investigated the potential molecular mechanisms that are involved in this antiapoptotic effect.

## 2. Materials and Methods

### 2.1. Materials

Allicin injection (5 mg/ml) was obtained from Xinjiang Ailexin Pharmacy Co., Ltd. (Urumqi, China). NG-nitro-l-arginine methyl ester (l-NAME), a specific pharmacological blocker of nitric oxide (NO), was purchased from Sigma (St. Louis, MO, USA). Anti-*β*-actin antibody was purchased from Beijing Zhongshan Golden Bridge Biotechnology (Beijing, China). Antibodies against Bax and Bcl-2 were purchased from Cell Signaling Technology (Danvers, MA, USA). Antibodies against endothelial nitric oxide synthase (eNOS), nuclear factor erythroid 2-related factor 2 (Nrf2), and heme oxygenase-1 (HO-1) were purchased from Abcam (Cambridge, UK). Horseradish peroxidase- (HRP-) conjugated anti-mouse and anti-rabbit immunoglobulin G antibodies were purchased from Beijing Zhongshan Golden Bridge Biotechnology (Beijing, China).

### 2.2. H9c2 Cell Culture and Ischemia/Hypoxia Model

H9c2 cells were cultured in Dulbecco's modified Eagle's medium (DMEM) that contained 10% fetal bovine serum, 2 mM glutamine, and antibiomycin (10 mM penicillin G and 10 mM streptomycin) at 37°C in a humidified 5% CO_2_ atmosphere and subcultured to approximately 80–90% confluence preexperimentally.

An I/H model was established to induce apoptosis. The culture medium was replaced with serum-free DMEM, and then H9c2 cells were transferred to a hypoxia chamber that was controlled by a ProOxC system balanced with 5% CO_2_/95% N_2_ at 37°C (Biospherix, Redfield, NY). The O_2_ concentration was ≤1%. The cells were pretreated with allicin at different concentrations (0.2, 1, and 5 *μ*M) for 1 h and then exposed to I/H for 12 h. The negative control cells were cultured in DMEM containing 10% fetal bovine serum without allicin.

### 2.3. Cell Viability Assay

Cell viability was examined using a 3-(4,5-dimethylthiazol-2-yl)-2,5-diphenyltetrazolium bromide (MTT) assay kit (Promega, Madison, WI, USA) according to the manufacturer's protocol. H9c2 cells were plated in 96-well plates at a density of 1 × 10^4^ cells/well. The cells were pretreated with various doses of allicin (0.2, 1, and 5 *μ*M) for 1 h and then exposed to I/H for 12 h. Afterward, a 100 *μ*l cell suspension was incubated with 20 *μ*l of CellTiter 96® AQueous One Solution Reagent for 1 h with 5% CO_2_ at 37°. Absorbance was read at 490 nm. The mean optical density (OD) of five wells was used to calculate the percentage of cell viability: % cell viability = OD_treatment group_/OD_control group_ × 100%.

### 2.4. Flow Cytometry Assay

The rate of apoptosis was determined by flow cytometry after staining the cells with Annexin V-FITC/PI (key GEN Bio TECH, Nanjing, China) according to the manufacturer's protocol. The cells were cultured in six-well plates at a density of 1 × 10^5^ cells/well, treated with allicin (0.2, 1, and 5 *μ*M) for 1 h, and then exposed to I/H for 12 h. The cells were harvested, washed twice with cold 1x phosphate-buffered saline (PBS), centrifuged at 2000 rotations per minute for 6 min, and resuspended in 0.5 ml of cold 1x binding buffer. The cell suspension was stained with 5 *μ*l of Annexin V-FITC for 10 min in the dark, and 5 *μ*l of propidium iodide (PI) was then added for 5–15 min in the light at room temperature. Flow cytometry was performed using a fluorescence-activated cell sorting instrument. The data were analyzed using WinMDI/PC software.

### 2.5. Measurement of Calcium Ion Concentrations

The cells were pretreated with various doses of allicin (0.2, 1, and 5 *μ*M) for 1 h and then subjected to I/H for 12 h. The cells were then incubated with Fluo-3/AM (Molecular Probes, USA) for 30 min at 37°C and then observed under a confocal laser scanning microscope.

### 2.6. Measurement of Oxidative Activity

The content of NO and malondialdehyde (MDA) and activity of sodium oxide dismutase (SOD) were assessed using specific kits (Nanjing Jiancheng Biological Engineering Institute, Nanjing, China). All of the procedures were performed according to the manufacturer's protocols.

### 2.7. Western Blot

Cell lysates (20 *μ*g of protein) were analyzed by 12% sodium dodecyl sulfate-polyacrylamide gel electrophoresis and electrotransferred to polyvinylidene difluoride membranes. The membranes were blocked with 5% bovine serum albumin and then probed with specific antibodies at 4°C overnight. After three washes in Tris PBS (TPBS), the membranes were incubated with HRP-conjugated secondary antibodies, followed by electrochemiluminescent detection. Blot densitometry was then performed. The bands were analyzed using a Gene Genius Bio Imaging System.

### 2.8. Statistical Analyses

The data were analyzed using SPSS 19.0 software. The values are expressed as mean ± SEM. Analysis of variance (ANOVA) was conducted, followed by Bonferroni correction, to test for differences in mean values between groups. The results were considered significant at *p* < 0.05.

## 3. Results

### 3.1. Allicin Improves H9c2 Cell Morphology and Viability

**Figure 1:**
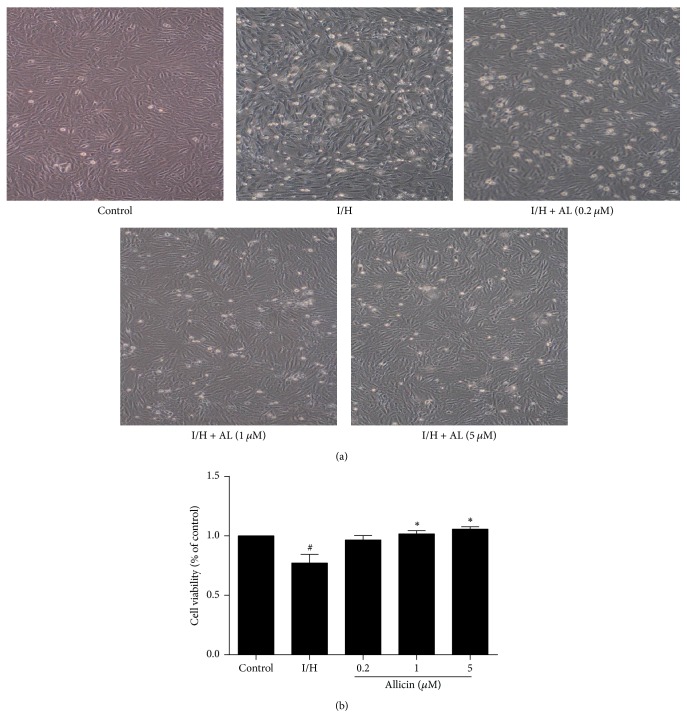
*Cell morphology (40x magnification) and viability in H9c2 cells that were* pretreated with allicin and subjected to I/H, evaluated in the MTT assay. (a) Representative images of H9c2 cells of the five groups during I/H. (b) Effect of allicin on cell viability. Five groups were evaluated: one group of control cells that were not pretreated with allicin and not subjected to I/H, one group of untreated H9c2 cells that were subjected to I/H, and three groups of H9c2 cells that were pretreated with 0.2, 1, and 5 *μ*M allicin and subjected to I/H. *n* = 3 independent experiments. The data are expressed as mean ± SEM. ^#^*p* < 0.05, compared with control; ^*∗*^*p* < 0.05, compared with I/H. I/H, ischemia/hypoxia; AL, allicin.

Morphological variations of H9c2 cells were observed under an inverted microscope. In the I/H group, the cells were severely damaged, with edge blurring, shrunken, shedding, and floating in the cell culture medium. In the allicin-pretreated groups, the degree of cell injury decreased, and cellular survival increased, especially in the group that was pretreated with 5 *μ*M allicin (Figure 1(a)).

Cell viability was examined using the MTT assay kit. Cell viability in the I/H group significantly decreased compared with the control (*p* < 0.05). Cell viability significantly increased in the groups that were treated with 1 and 5 *μ*M allicin compared with the untreated I/H group (*p* < 0.05). No significant difference (*p* > 0.05) was observed between the group that was treated with 0.2 *μ*M allicin and the untreated I/H group (Figure 1(b)).

### 3.2. Allicin Decreases Rate of I/H-Induced Apoptosis in H9c2 Cells

**Figure 2:**
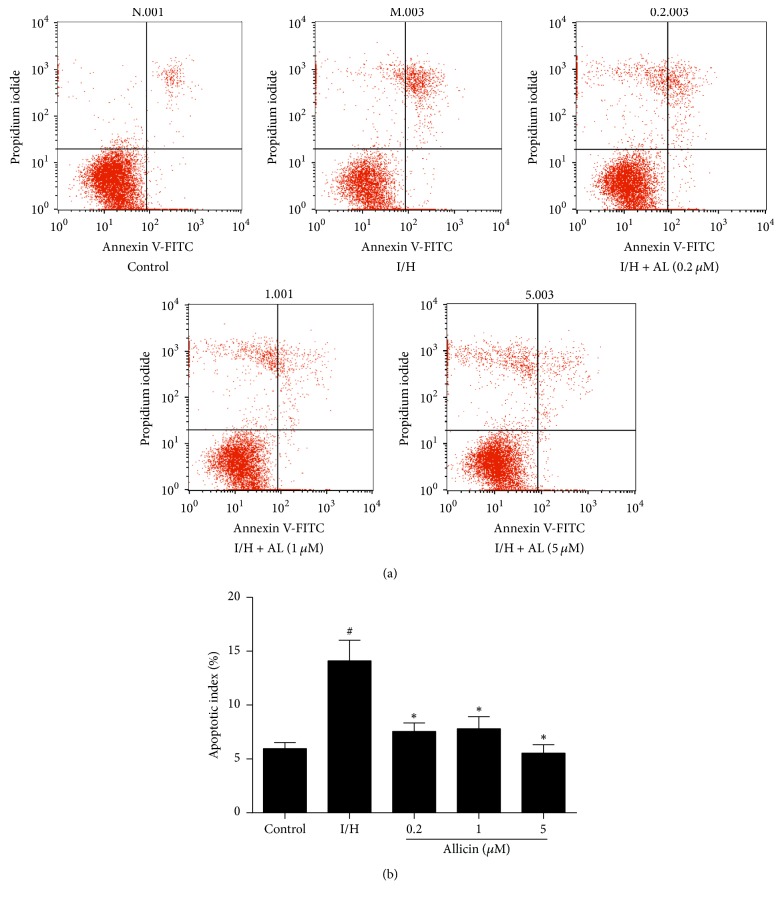
*Effect of allicin on rate of I/H-induced apoptosis in H9c2 cells, assessed by flow cytometry.* (a) Representative images of H9c2 cell apoptosis during I/H. (b) Effect of allicin on rate of apoptosis. Five groups were evaluated: one group of control cells that were not pretreated with allicin and not subjected to I/H, one group of untreated H9c2 cells that were subjected to I/H, and three groups of H9c2 cells that were pretreated with 0.2, 1, and 5 *μ*M allicin and subjected to I/H. *n* = 3 independent experiments. The data are expressed as mean ± SEM. ^#^*p* < 0.05, compared with control; ^*∗*^*p* < 0.05, compared with I/H. I/H, ischemia/hypoxia; AL, allicin.

After incubation under conditions of I/H for 12 h, the rate of apoptosis was determined by flow cytometry after staining the cells with Annexin V-FITC/PI. In the untreated I/H group, apoptosis significantly increased compared with the sham control group (*p* < 0.05). Apoptosis in the allicin-treated groups significantly decreased compared with the untreated I/H group (*p* < 0.05; Figure 2).

### 3.3. Allicin Decreases Intracellular Ca^2+^ Concentrations

**Figure 3:**
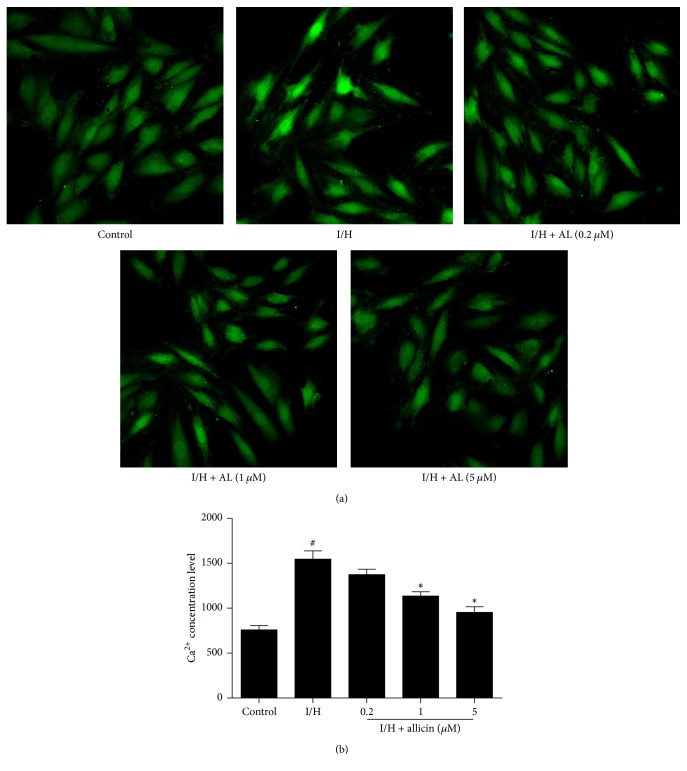
*Effect of allicin on intracellular Ca* ^*2+*^ *concentrations during I/H.* (a) Representative images of intracellular Ca^2+^ concentrations in H9c2 cells during I/H. (b) Effect of allicin on intracellular Ca^2+^ concentrations. Five groups were evaluated: one group of control cells that were not pretreated with allicin and not subjected to I/H, one group of untreated H9c2 cells that were subjected to I/H, and three groups of H9c2 cells that were pretreated with 0.2, 1, and 5 *μ*M allicin and subjected to I/H. *n* = 3 independent experiments. The data are expressed as mean ± SEM. ^#^*p* < 0.05, compared with control; ^*∗*^*p* < 0.05, compared with I/H. I/H, ischemia/hypoxia; AL, allicin.

To detect intracellular Ca^2+^ concentrations, the cells were incubated with Fluo-3/AM and then observed under a confocal laser scanning microscope. Ca^2+^ concentrations in the untreated I/H group significantly increased compared with the control group (*p* < 0.05; Figure 3). In the allicin-treated groups, a significant decrease in Ca^2+^ concentrations was observed compared with the untreated I/H group (*p* < 0.05).

### 3.4. Allicin Suppresses the Expression of Markers of Apoptosis

**Figure 4:**
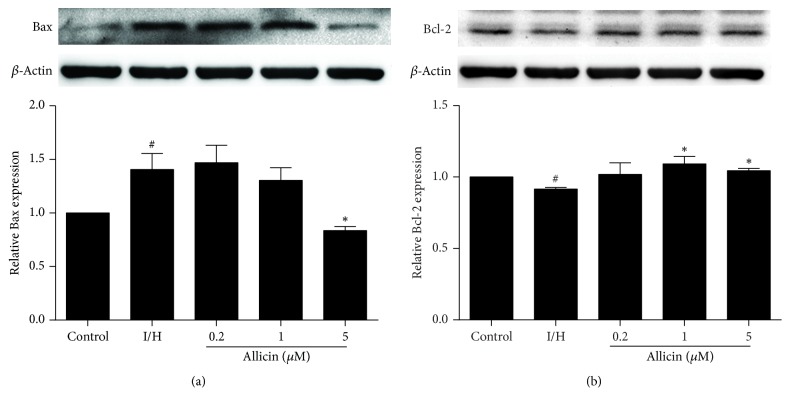
*Effect of allicin on Bax and Bcl-2 expression in H9c2 cells, evaluated by Western blot.* (a) Allicin decreased Bax expression. (b) Allicin increased Bcl-2 expression. Five groups were evaluated: one group of control cells that were not pretreated with allicin and not subjected to I/H, one group of untreated H9c2 cells that were subjected to I/H, and three groups of H9c2 cells that were pretreated with 0.2, 1, and 5 *μ*M allicin and subjected to I/H. *n* = 3 independent experiments. The data are expressed as mean ± SEM. ^#^*p* < 0.05, compared with control; ^*∗*^*p* < 0.05, compared with I/H. I/H, ischemia/hypoxia; AL, allicin.

Bax and Bcl-2 are markers of apoptosis. Bax and Bcl-2 protein expression was detected by Western blot. Bax expression significantly increased and Bcl-2 expression significantly decreased in H9c2 cells that were subjected to I/H compared with the control group. Bax expression significantly decreased in the group that was pretreated with 5 *μ*M allicin, and Bcl-2 expression significantly increased in the groups that were pretreated with 1 and 5 *μ*M allicin (*p* < 0.05; Figures 4(a) and 4(b)).

### 3.5. Allicin Increases eNOS Expression and NO Levels

**Figure 5:**
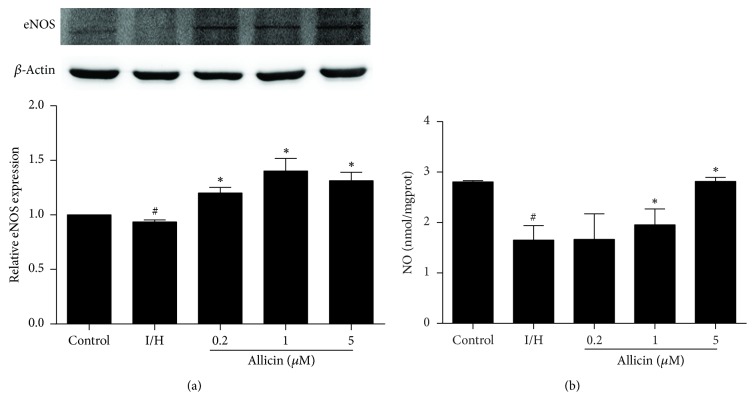
*Effect of allicin on eNOS expression and NO levels in H9c2 cells.* (a) Allicin increased eNOS expression. (b) Allicin increased NO levels. Five groups were evaluated: one group of control cells that were not pretreated with allicin and not subjected to I/H, one group of untreated H9c2 cells that were subjected to I/H, and three groups of H9c2 cells that were pretreated with 0.2, 1, and 5 *μ*M allicin and subjected to I/H. *n* = 3 independent experiments. The data are expressed as mean ± SEM. ^#^*p* < 0.05, compared with control; ^*∗*^*p* < 0.05, compared with I/H. I/H, ischemia/hypoxia; AL, allicin.

Nitric oxide is a potent gaseous signaling molecule. eNOS-derived NO may participate in the pathophysiological regulation of ischemic heart disease. We detected eNOS expression and NO levels that were induced by I/H in H9c2 cells. eNOS expression significantly decreased in untreated cells that were subjected to I/H compared with the control group (*p* < 0.05). In the allicin-pretreated groups (0.2, 1, and 5 *μ*M), eNOS expression significantly increased compared with the untreated I/H group (*p* < 0.05; Figure 5(a)). NO levels significantly decreased in untreated cells that were subjected to I/H compared with the control group. In cells that were treated with allicin (1 and 5 *μ*M), NO levels dose-dependently increased (*p* < 0.05; Figure 5(b)).

### 3.6. Effect of L-NAME on Antiapoptotic Effect of Allicin

**Figure 6:**
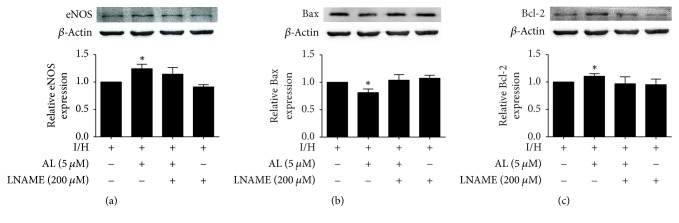
*Effect of allicin and* l-*NAME on the expression of (a) eNOS, (b) Bax, and (c) Bcl-2 in H9c2 cells, evaluated by Western blot.* Four groups were evaluated: one group of H9c2 cells that were not treated with either allicin or l-NAME and were subjected to I/H, one group of H9c2 cells that were pretreated with 5 *μ*M allicin and subjected to I/H, one group of H9c2 cells that were pretreated with 5 *μ*M allicin and 200 *μ*M l-NAME and subjected to I/H, and one group of H9c2 cells that were pretreated with 200 *μ*M l-NAME and subjected to I/H. *n* = 3 independent experiments. The data are expressed as mean ± SEM. ^*∗*^*p* < 0.05, compared with I/H. I/H, ischemia/hypoxia; AL, allicin.

We used l-NAME, a specific pharmacological blocker of NO, to elucidate the mechanism of action of allicin. l-NAME significantly inhibited eNOS expression (*p* < 0.05; Figure 6(a)) and inhibited the cardioprotective effect of allicin against apoptosis, reflected by an increase in Bax expression and decrease in Bcl-2 expression (*p* < 0.05; Figures 6(b) and 6(c)).

### 3.7. Effect of Allicin on Oxidative Activity in H9c2 Cells

**Figure 7:**
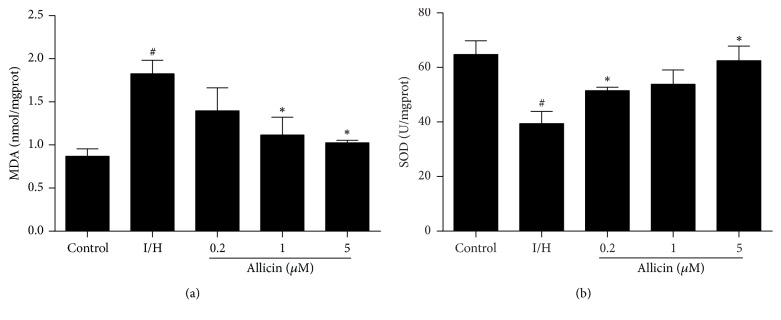
*Effects of allicin on MDA and SOD levels in H9c2 cells.* (a) Allicin downregulated MDA levels in H9c2 cells. (b) Allicin increased SOD activity in H9c2 cells. Five groups were evaluated: one group of control cells that were not pretreated with allicin and not subjected to I/H, one group of untreated H9c2 cells that were subjected to I/H, and three groups of H9c2 cells that were pretreated with 0.2, 1, and 5 *μ*M allicin and subjected to I/H. *n* = 3 independent experiments. The data are expressed as mean ± SEM. ^#^*p* < 0.05, compared with control; ^*∗*^*p* < 0.05, compared with I/H. I/H, ischemia/hypoxia; AL, allicin.

Malondialdehyde and SOD are biomarkers of oxidative stress. To determine whether allicin functions at the level of oxidative stress, we measured MDA and SOD in H9c2 cells. Our results showed that MDA levels significantly increased and SOD activity significantly decreased during I/H, and these effects were reversed by allicin pretreatment (Figures 7(a) and 7(b)).

### 3.8. Effect of Allicin on Nrf2 and HO-1 Expression in H9c2 Cells

**Figure 8:**
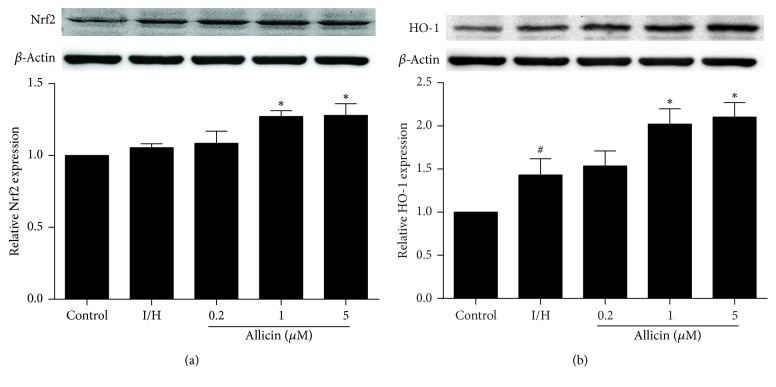
*Effect of allicin on Nrf2 and HO-1 expression in H9c2 cells, evaluated by Western blot.* (a) Allicin increased Nrf2 expression. (b) Allicin increased HO-1 expression. Five groups were evaluated: one group of control cells that were not pretreated with allicin and not subjected to I/H, one group of untreated H9c2 cells that were subjected to I/H, and three groups of H9c2 cells that were pretreated with 0.2, 1, and 5 *μ*M allicin and subjected to I/H. *n* = 3 independent experiments. The data are expressed as mean ± SEM. ^#^*p* < 0.05, compared with control; ^*∗*^*p* < 0.05, compared with I/H. I/H, ischemia/hypoxia; AL, allicin.

We further examined whether allicin affects activation of the Nrf2/HO-1 signaling pathway. I/H induced a trend toward higher Nrf2 expression in H9c2 cells (*p* > 0.05). Treatment with allicin (1 and 5 *μ*M) significantly increased Nrf2 expression (*p* < 0.05; Figure 8(a)). Moreover, I/H significantly increased HO-1 expression in H9c2 cells. Treatment with allicin (1 and 5 *μ*M) induced a further dose-dependent increase in HO-1 expression (*p* < 0.05; Figure 8(b)).

## 4. Discussion

Allicin is one of the critical bioactive organosulfur compounds in garlic. It has been reported to have a number of bioactivities including antioxidant, cardioprotective activity [15, 16]. However, due to its unstable nature, allicin is rapidly degraded with time depending on environmental and processing conditions, such as temperature, light, and concentration [17–19]. Document has also shown that the biological half-life of allicin is significantly longer in alcoholic and aqueous extracts than the chemical one [20]. In the present study, allicin is a water-soluble injection and stored in dark place at 4°C to ensure its stability. Then, we assessed the antiapoptotic effect of allicin in H9c2 cells. Allicin significantly decreased intracellular calcium concentration and the rate of apoptosis. Meanwhile, allicin significantly increased Bax expression and decreased Bcl-2 and eNOS expression. However, the NO blocker l-NAME partially reversed these beneficial effects of allicin, indicating an involvement of eNOS/NO signaling pathway. Allicin also increased SOD activity, NO release, and Nrf2 and HO-1 expression and decreased MDA levels. Altogether, these findings suggest that activation of the eNOS/NO pathway by allicin and its antioxidant and antiapoptotic effects play an important role in its cardioprotective effects.

Apoptosis is a key regulator in the pathogenesis of myocardial ischemia. Bcl-2 family members act upstream of mitochondrial-mediated apoptosis and play a central role in cell fate and homeostasis. Bcl-2 (prosurvival) protein expression determines whether the cell undergoes apoptosis or reenters the cell cycle. Bax protein expression integrates important functions that are related to apoptosis and facilitates the release of cytochrome c from mitochondria [21, 22]. Therefore, the ratio of Bcl-2/Bax expression is regarded as a hallmark in cell survival or death upon apoptotic stimulation [23, 24]. In the present study, we utilized I/H method to induce apoptosis in H9c2 cells, and allicin significantly increased cell activity and decreased the rate of apoptosis that was induced by I/H, reflected by a decrease in Bax expression and increase in Bcl-2 expression. Cellular injury caused I/H is also accompanied by intracellular calcium overload. Increasing intracellular Ca^2+^ contributes to the development and progression of myocardial apoptosis [25]. Our results showed that intracellular calcium concentrations significantly decreased in the allicin-pretreated groups.

Nitric oxide is a potent gaseous signaling molecule that is synthesized by a family of NOS enzymes, including inducible, neuronal, and endothelial forms [26]. eNOS, also known as NOS III, is a low-output enzyme that is constitutively expressed in H9c2 cells. eNOS-derived NO has been reported to participate in the pathophysiology of ischemic heart disease, such as myocardial infarction and myocardial ischemia-reperfusion injury [27, 28]. A previous study reported that both skin and flesh garlic extracts effectively prevented norepinephrine-induced cardiomyocyte hypertrophy and cell death, and these beneficial effects were partially mediated by NO and H_2_S [29]. Our previous findings showed that plasma H_2_S levels dose-dependently increased in allicin-treated rats, indicating that allicin may be an H_2_S donor [13]. The present study demonstrated the involvement of NO in this process. Allicin treatment increased eNOS protein expression and NO levels. To further explore the mechanism by which allicin attenuates myocardial cell apoptosis, we pharmacologically blocked NO using l-NAME. l-NAME significantly inhibited the cardioprotective effect of allicin against apoptosis, evidenced by an increase in Bax expression and decrease in Bcl-2 expression. These data strongly suggest that the eNOS/NO pathway is involved in the effects of allicin against apoptosis.

Oxidative stress promotes cell death in response to various pathophysiological conditions. Reaction oxygen species (ROS) are free radicals, the accumulation of which can cause oxidative stress that damages the heart in myocardial ischemia [30]. Previous studies reported that allicin protected cells against oxidative stress by inhibiting the generation of intracellular ROS [31, 32]. Nrf2 is a redox-sensitive transcription factor that plays a key role in cellular antioxidant defense [33]. In the presence of oxidative stress, Nrf2 is rapidly degraded by the proteasome system, enters the nucleus, binds to antioxidant response element, and upregulates multiple antioxidant and detoxifying genes, such as HO-1 [34]. Allicin was previously reported to prevent the development of cardiac remodeling and progression of cardiac hypertrophy to cardiac dysfunction by enhancing Nrf2 antioxidant signaling pathways [35]. A reasonable speculation is that the antiapoptotic effect of allicin may be associated with activation of the Nrf2 signaling pathway associated with oxidative stress. Therefore, we tested the effects of allicin on oxidative stress. Allicin significantly decreased MDA levels and increased SOD activity. It also promoted Nrf2 synthesis and nuclear translocation and further increased the expression of HO-1 protein, indicating the antioxidative activity of allicin in our I/H model. Numerous studies have shown that NO can scavenge ROS and attenuate the detrimental effects of ROS [36, 37]. In the present study, allicin increased eNOS protein expression and NO levels in H9c2 cells, and the NO blocker l-NAME significantly inhibited the cardioprotective effect of allicin against apoptosis. Therefore, we suggest that the antioxidative activity of allicin may be linked to activation of the eNOS/NO pathway.

## 5. Conclusion 

Overall, our data indicate that allicin has powerful protective effects against I/H-induced cell apoptosis. The mechanism appears to involve an antioxidative effect that is mediated by the eNOS/NO pathway. Allicin treatment may be a promising clinical approach for IHD.
